# Options in Targeted Therapy for Advanced Cholangiocarcinoma: A 2024 Update

**DOI:** 10.7759/cureus.59793

**Published:** 2024-05-07

**Authors:** Anca Monica Oprescu Macovei, Dana Paula Venter, Gratiella Georgiana Makkai, Sebastian Valcea, Mircea Dan Venter, Adrian Tulin, Mihai Stefan, Oprescu Constantin

**Affiliations:** 1 Gastroenterology, Agrippa Ionescu Emergency Clinical Hospital, Bucharest, ROU; 2 Pediatric Surgery, Grigore Alexandrescu Emergency Pediatric Hospital, Bucharest, ROU; 3 General Surgery, Floreasca Emergency Clinical Hospital, Bucharest, ROU; 4 General Surgery, Agrippa Ionescu Emergency Clinical Hospital, Bucharest, ROU; 5 Surgery, Bucharest Emergency Hospital, Bucharest, ROU

**Keywords:** medicine, oncology, cancer, genetics, gastroenterology

## Abstract

Bile duct carcinomas have a different prognosis and genetic profile depending on their location; intrahepatic/extrahepatic or at the level of the gallbladder. Although in recent years there have been important advances in first-line therapy, second-line therapy in cholangiocarcinoma does not currently have a standard. Therefore at this level, there is an acute need for personalized treatment. The present article is a narrative review that aims to list the newest targeted therapeutic options for this type of cancer, based on identified genetic alterations. The literature selected for analysis includes phase 2 or 3 studies with targeted therapy in this disease and original articles no older than three years that describe the prevalence of the most common gene alterations in this type of cancer. PubMed/Medline, Scopus, and Clarivate-Web of Science databases were searched and keywords such as "cholangiocarcinoma," "biliary cancer," "targeted therapy," "gene amplifications," and "mutations" were used. This narrative review was designed taking into account the SANRA (Scale for the Assessment of Narrative Review Articles) criteria. The conclusions lead to the fact that next-generation sequencing testing is of particular usefulness in cholangiocarcinoma. Bile duct cancers are rich in targetable genetic alterations, and their treatment is in constant change, although much of the current data comes from phase II studies. There is a great need for the current options to be analyzed in phase III studies. Hence, the need of the oncological community to stay informed about targeted treatment options for cholangiocarcinoma is supported by the present article.

## Introduction and background

Bile duct carcinomas have a different prognosis and genetic profile depending on their location; intrahepatic/extrahepatic or at the level of the gallbladder. Although in recent years there have been important advances in first-line therapy in this location, with the addition of immunotherapy to chemotherapy as the standard first-line, the benefit on overall survival remains modest. One of the most recent real-world analyses on overall survival with this regimen in the first line for cholangiocarcinoma reported only 12.8 months median survival [1]. Comparable median overall survival was found in the phase 3 TOPAZ-1 trial, which established this regimen as standard of care (durvalumab+gemcitabine+cisplatin 12.8 months, 11.1-14.0, odds ratio=0.8, versus placebo+gemcitabine+cisplatin 11.5 months) [2]. The second-line therapy in cholangiocarcinoma is not routine practice, and usually, clinicians prefer targeted treatment if available. Cholangiocarcinoma, although rare (approximately 3% of all gastroenterological malignancies) [3] is a fatal disease with a median overall survival of less than one year and a five-year survival of less than 5% [3]. The incidence of cholangiocarcinoma differs by region, with the highest incidence in northwestern Thailand (85 cases per 100,000 inhabitants), followed by central Thailand (14 per 100,000 inhabitants) [4]. In Europe, the highest incidence was reported in Italy (3.4 cases per 100,000) [3]. Gene alterations involving IDH (isocitrate dehydrogenase gene), FGFR (fibroblast growth factor receptor), BRAF (v-RAF murine sarcoma viral oncogene homolog B1),HER2 (human epidermal growth factor receptor 2) can decisively influence the proliferation and development of cholangiocarcinoma [5]. Along with the use of immune checkpoint inhibitors in this location, the need to identify effective predictive biomarkers also appeared, but research in this area is still ongoing. Some of the earliest analyses of the targeted treatment options for cholangiocarcinoma stated that 68% of the cholangiocarcinoma specimens carry actionable mutations [6]. When searching clincialtrials.gov with biliary tract cancer as a condition/disease and using metastatic for other terms, 311 studies were listed, with 69 recruiting, 14 not yet recruiting, 29 active but not recruiting, 122 completed, and 29 terminated [7]. The present article is a narrative review that aims to list the newest targeted therapeutic options for this type of cancer, based on identified genetic alterations.

## Review

Search strategy and selection criteria

This narrative review was designed taking into account the SANRA (Scale for the Assessment of Narrative Review Articles) criteria and is based on recent publications on targeted therapies in advanced cholangiocarcinoma. No committee approval was required for the article [3]. The literature selected for presentation included phase 2 or 3 studies with targeted therapy in this disease and original articles no older than three years that describe the prevalence of the most common gene alterations in this type of cancer. PubMed/Medline, Scopus, and Clarivate-Web of Science databases were searched and keywords such as "cholangiocarcinoma," "biliary cancer," "targeted therapy," "gene amplifications," and "mutations" were used. Publications such as notes, commentaries or conference proceedings, or publications in a language other than English were excluded. The literature selection was made separately by two of the authors, the list of publications being compared for an optimal and unbiased selection. A number of articles were thus included and analyzed.

Genetic alterations frequency

IDH1mutations are relatively common in patients with intrahepatic tumors. A study that enrolled 5393 patients with cholangiocarcinoma reported a frequency of 13.1% in this location, as opposed to 0.8% in patients who had extrahepatic tumors [8]. There was no information related to the frequency of mutations according to gender and age. Also, their prevalence was lower in the Asian population compared to the non-Asian population [8]. FGFR alterations were most frequently identified in urothelial, breast, endometrial, squamous lung, and ovarian carcinomas. Cholangiocarcinomas most often present FGFR fusions, especially fusions of the second receptor, and are frequently manifested in intrahepatic tumors (16%). Extrahepatic cholangiocarcinoma most often has amplifications (2.6%) and activating mutations (0.9%) [9]. HER2 overexpression identified by immunohistochemistry is identified in 50% of bile duct carcinomas (5% in extrahepatic bile duct carcinomas, 16% in gallbladder carcinomas, and 12% in ampulla of Vater carcinomas). They are rarely present in intrahepatic bile duct carcinomas. Among the bile duct carcinoma patients who performed next-generation sequencing (NGS) testing, 5.4% report gene alterations involving HER2, of which 2.4% amplifications, 2.3% ERBB2 mutations, and 0.4% with mutations and amplifications present simultaneously [9]. Gallbladder tumors were the most representative of this type of alteration, at 12.6%. In addition to HER2 gene alterations, others of interest were simultaneously identified for the patients, such asTP53 gene-54%, PIK3CA gene 21%, and MSI-H (microsatellite instability biomarker) at 7% [10]. BRAF gene mutation occurs in 5-7% of bile duct carcinomas, with a higher frequency in intrahepatic ones, the most frequent mutation in this case being BRAF 600E [11]. The frequency of EGFR (epidermal growth factor receptor) expression in bile duct carcinomas is different depending on the analysis method. In the FISH (fluorescence in situ hybridization) analysis, 46% of the analyzed samples recorded an increased number of EGFR gene copies. When the samples were analyzed by immunohistochemistry, EGFR 2+/3+ was identified in 59% of cases with a prevalence of 68%, 58%, and 33% respectively for extrahepatic bile ducts, intrahepatic bile ducts, and gallbladder carcinomas [11]. OverexpressedEGFR is also considered a negative prognostic factor, with five-year survival being 20% in those with this genetic alteration versus 60% in patients without overexpression [12]. Kendre et al. aimed to analyze the genomic heterogeneity of biliary carcinoma in 2022, analyzing co-mutation patterns associated with targetable drivers [13]. Thus, the authors proposed to retrospectively analyze 6130 patients with this location which were included in the FoundationCORE database. A representative image of the genomic landscape resulted, which is summarized in Table 1 [13].

**Table 1:** Frequency of the most prevalent alterations per cholangiocarcinoma location. [13] IHC: intrahepatic cholangiocarcinoma, ECC: extrahepatic cholangiocarcinoma, IDH: isocitrate dehydrogenase, FGFR: fibroblast growth factor receptor, KRAS: Kirsten rat sarcoma virus, TP53: tumor suppressor 53, MDM2: mouse double minute 2, CDKN2A/B: cyclin-dependent kinase N2A/B.

Genetic alteration	IHC	Gallblader	ECC
IDH	14 %	2%	3%
FGFR	11%	3%	5%
BRAF	5%	1%	5%
KRAS	20%	11%	36%
TP53	33%	63%	52%
MDM2	4%	0	6%
CDKN2A/B	30%	21%	32%

Immunotherapy biomarkers

The current standard of first-line therapy in advanced biliary carcinoma is represented by gemcitabine and cisplatin in combination with durvalumab or pembrolizumab. Although biliary carcinoma is considered a cancer with a cold immunogenic microenvironment, both trials demonstrated the effectiveness of the addition of immune checkpoint inhibitors probably due to the immunogenic effect of chemotherapy [14,15]. However, there are biomarkers that can predict a good response to immune checkpoint inhibitors. The most important are TMB (tumor mutational burden) and microsatellite instability. There is evidence from studies on the Asian population that the incidence of chromosomal instability is low in biliary carcinoma regardless of its location (18%), and less than 3% of patients with this histology have TMB over 15 mutations/Mb [16]. Alterations in DNA repair genes such as ATM, BRCA1/2, MLH1, MSH2, PALB2, POLE and caretaker genes *TP53 *and *BAP1 *seem to be in greater numbers in patients with gallbladder carcinomas (14% and 63%, respectively) [17]. These can be considered biomarkers of favorable response to immune checkpoint inhibitors, although the implications can be much more complex. For example, mutations at the level of the POLE gene or EDM gene can be associated with ultra-mutated or stable microsatellite phenotype [17,18].

Targetable alterations

Isocitrate Dehydrogenase 1

IDH proteins 1 and 2 from the IDH gene are essential in DNA repair and epigenetic regulation, playing a fundamental role in inhibiting homologous repair mechanisms. If mutations occur within them, enzyme activity is distorted, alpha-ketoglutarate can be converted to 2-hydroxyglutarate, inhibiting the activity of multiple ketoglutarate-dependent enzymes. As a result, DNA can no longer be repaired, and histone demethylation remains deficient [19]. The most common gene alterations involving IDH1 are fusions, more frequent in intrahepatic cholangiocarcinoma, followed by amplification and activating mutations, found in extrahepatic tumors. Given their prevalence and the fact that there is a targeted therapeutic option for them, several clinical trials have been conducted in recent years to verify their effectiveness [20]. Ivosidenib and olutasidenib are the most widely used and known IDH1 inhibitors, ivosidenib being the first studied in cholangiocarcinoma in a phase 1 study, with encouraging results [21]. Subsequently, the ClarIDHy phase III study enrolled 187 patients with advanced cholangiocarcinoma with IDH1 mutation, who progressed after one or two treatment regimens. They were randomized 2:1 to ivosidenib 500 mg per day (n=126) versus placebo (n=61). The data showed superiority regarding progression-free survival (2.7 months versus 1.4 months, HR=0.37 p<0.0001), and crossover was permitted. The treatment was well tolerated, the most frequent adverse reactions being cardiovascular, dermatological, and hydroelectrolytic disorders. No deaths due to treatment were reported. Quality of life was maintained in patients receiving studied treatment [22]. Data related to overall survival were published in 2021. The maximum duration of treatment with ivosidenib was 34.4 months and 6.9 months with placebo (we have to take into account the high cross-over rate data at 70%). Overall survival was 10.3 months with ivosidenib (95% CI=4.8-11.1 months) versus 7.5 months with placebo (HR=0.79, 95% CI=0.56-1.12) [23].

*Fibroblast Growth Factor Receptor* 

**Figure 1:**
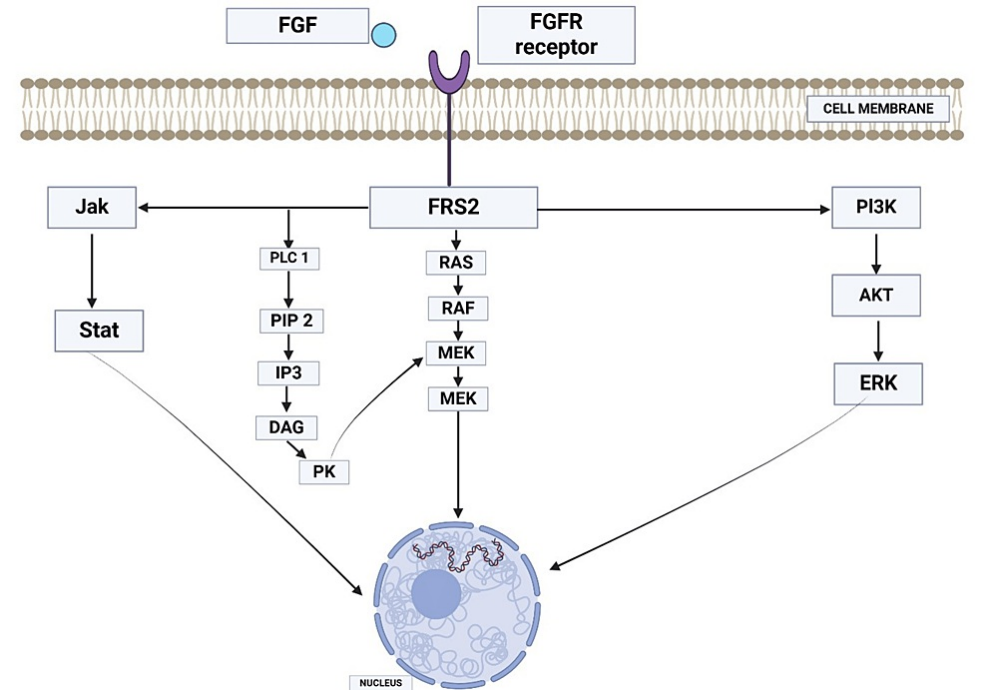
The FGF pathway in brief. After FGF (fibroblast growth factor) binds to the transmembrane receptor, FGFR (fibroblast growth factor receptor) dimerizes and activates the intracellular pathways. Through the JAK/STAT (Janus kinase/signal transduction and transcription activation), RAS (rat sarcoma virus)/RAF (rapidly accelerated fibrosarcoma)/MEK (mitogen-activated extracellular signal-regulated kinase) and PI3K (phosphoinositide 3-kinase)/Akt (protein kinase B)/ERK pathways (extracellular signal-related kinase), DNA transcription is activated and consequently cell proliferation, migration, angiogenesis, and DNA repair take place. Picture generated at BioRender.com.

The family of transmembrane receptors of fibroblast growth factors (FGFR) includes four members whose mediators are fibroblast growth factors. They have an intracellular tyrosine kinase correspondent that conducts signaling to regulate angiogenesis, migration, DNA repair, and cell proliferation. Their activity is essential in both developing and adult cells [24]. Being so important functionally and being involved in the appearance of a large number of neoplasms, they were considered attractive therapeutic targets [24]. TheFGFR signaling pathway can be summarized in Figure 1.

Pemigatinib, an inhibitor of FGFR 1, 2, and 3, has been evaluated since for its effectiveness in cholangiocarcinoma. The phase II FIGHT-202 study enrolled 146 patients, 107 with FGFR fusions and rearrangements, 20 with other gene alterations involving this receptor, 18 without gene alterations of this type, and one with FGFR alteration of undetermined significance. All patients received pemigatinib 13.5 mg orally, daily, in a 21-day regimen (treatment for 14 days and a one-week break). The response rate was worthy of consideration (35.5% [95%CI 26.5-45.4]), with three complete responses also recorded. The most frequent adverse reaction was hyperphosphatemia (which occurred in 60% of patients, with three in 10% of cases). Among patients, 45% have serious adverse events, including abdominal pain, fever, and cholangitis. There were no deaths due to the treatment [25]. After these promising results, it was proposed to test the effectiveness of pemigatinib in the first line, compared to gemcitabine and cisplatin in patients with advanced cholangiocarcinoma and FGFR fusions/rearrangements. This will be tested in the FIGHT 302 study, an ongoing phase III study. the primary endpoint is progression-free survival, and secondary endpoints include overall survival, response rate, safety, and quality of life. The study aims to enroll 432 patients to enroll them 1:1 for pemigatinib 13.5 mg daily, 14 days, in a 21-day regimen or standard of care [26]. Infigratinib, an ATP (adenosine triphosphate)-dependent FGFR inhibitor, is another therapeutic option tested in a phase II trial that enrolled 122 patients with FGFR2 fusions or rearrangements. They received the studied medication orally, in a dose of 125 mg per day, for 21 days with a 1-week break (28-day regimen). After a median follow-up of 10.6 months, the reported response rate was 23.1% (95% CI15.6-32.2, 25 of 108 patients) with a complete imaging response. Hyperphosphatemia was the most frequent adverse effect, followed by stomatitis, physical asthenia, and alopecia. Adverse ophthalmological effects have also been reported. There were no treatment-related deaths [27]. The LUC2001 study is a phase 2, multicenter open-label trial that verified the efficacy of erdafitinib in patients with cholangiocarcinoma and FGFR gene alterations who progressed on at least 1 line of treatment. Out of 232 screened patients, all Asian, 39 (16.8%) had FGFR alterations and 22 (9.5%) were considered eligible. The response rate was 40.9% (95% CI 20.7+63.6%) and the median time to respond was 1.8 months (range 1.5+5.6). As notable adverse events, the trial mentioned stomatitis and the increase of liver enzymes, thus concluding that erdafitinib is an option with a durable effect and manageable side effects [28]. Futibatinib is a selective, irreversible inhibitor of the signaling pathway involving FGFR. Its irreversible nature makes futibatinib not as susceptible to on-target resistance mutations as pemigatinib or infigratinib. It was studied in a phase 2 trial called FOENIX-CCA2, which included 103 patients who received 20 mg orally per day of the studied treatment. The response rate was 42%, n=43, 95% CI 32-52, and one patient had a complete response. The mean duration of response was 9.7 months (95% CI 7.6-17.0). Among the patients, 66% had no progression at six months and 40% had at least stationary disease at 12 months. Overall survival was 21.7 months (95% CI 14.5- not reached at the time of publication). Notable toxicities (with grades above 3) included hyperphosphatemia, increased transaminases, stomatitis, and fatigue [29].

Human Epidermal Growth Receptor 2

The human epidermal growth factor receptor, a protein encoded by the ERBB2 gene (located on chromosome 17) is essential in the regulatory mechanisms of cell proliferation. Its overexpression is considered a bad prognostic factor in any oncological location and is associated with shorter survival. HER2 amplification is therefore considered an oncogenic driver. Starting from the therapies studied in breast neoplasm, it was proposed to use them in other locations where it is frequently present, such as urothelial and bile duct carcinoma [30]. The combination of targeted anti-HER2 treatment and the standard of first-line treatment in metastatic bile duct carcinoma was studied in a phase II trial that enrolled 90 patients and received trastuzumab 8 mg/kg in the initial dose followed by 6 mg/kg at three weeks, together with cisplatin 25 mg/m2 and gemcitabine, days 1 and 8, q3w. The median progression-free survival was seven months (95% CI = 6.2-7.8 months). The progression-free survival rate at six months was 75.6%, exceeding the 60% limit that the study proposed, and the 12-month rate was 17.6%. The coexistence of p53 mutation with HER2 gene alterations was associated with a much-reduced survival. A response rate of 55.5% was recorded, and 22.4% of patients had stationary disease, so the disease control rate was 80% [31]. The MyPathway basket study verifies targeted therapies in patients who have targetable gene alterations, but for whom the respective treatments are not therapeutic standard [32]. The main aim of the study is to evaluate the response rate. It proposes the use of pertuzumab and trastuzumab in patients with HER2 amplifications, over-expressions, or mutations. A total of 346 patients with colorectal cancer (27.5%), non-small cell lung cancer (17.3%), biliary tract cancer (14.7%), and gynecological cancers (12.7%) were enrolled. The study concluded that the combination is effective in patients with HER2 amplification and overexpression if they have KRAS gene wild-type mutation and is not suitable for patients who have HER2 mutations or negative HER2 expression or 1+ on immunohistochemistry [32]. The combination of tucatinib and trastuzumab was proposed in patients with bile duct carcinoma with previous systemic treatment and HER2 receptor overexpression. A total of 30 patients were enrolled who received tucatinib 300 mg twice a day and trastuzumab in a loading dose of 8 mg/kg and 6 mg/kg, the study proposing to establish the response rate. The response rate was 47%, and the disease control rate was 77%. The average duration of response was six months. The study concludes that this combination is one with remarkable efficiency and an acceptable safety profile [33]. Zanidatamab is a bispecific anti-HER2 antibody, which binds additional molecules in trans and initiates particular HER2 reorganization with increased efficiency, greater in preclinical studies than that of trastuzumab combined or not with pertuzumab. It was studied in patients with metastatic or unresectable bile duct carcinoma, pretreated with gemcitabine in the HERIZON-BTC-01 study. A total of 87 patients who had 2+ or 3+ expression on IHC were included; they had a response rate of 41.2% with a median duration of response of 12.9 months [34]. The phase II study DESTINY-PanTumor02 verifies the effectiveness of fam-trastuzumab deruxtecan (T-DXd) in patients with non-breast cancer oncological locations with HER2 overexpression. It enrolled 267 patients with biliary carcinoma, urinary bladder, cervix, endometrial, ovarian, pancreatic, and several other locations. The patients had advanced or resectable local disease that had progressed after at least one line of standard treatment. Of these, 40% had HER2 3+ and 57% HER2 2+. The overall response rate was 37.1% with a response duration of 11.8 months. In patients with 3+ expression on immunohistochemistry, the response rate was 61.3%. Among the patients, 5.6% had a complete response. Among those with bile duct carcinomas, the overall response rate was 22% (56.5% for IHC 3+ and 0% for IHC 2+). This trial supports the use of T-DXd in HER2 3+ bile duct carcinomas [35].

BRAF Mutation

The BRAF gene encodes a cytoplasmic kinase with a role in signaling during cell division. The mutation at its level involves the substitution of the amino acid valine with glutamic acid in codon 600 and involves the monomeric activation of BRAF and the activation of the MAPK (mitogen-activated protein kinases) signaling pathway [36]. The first basket study with patients with tumors with BRAF V600E mutation that also included patients with bile duct cancer was called the Rare Oncology Agnostic Research (ROAR) and was published in 2020. A total of 43 patients were enrolled in the subgroup with bile duct cancer, these being in advanced local non-resectable or metastatic stage and with at least one prior line of treatment. The independent reviewer assessed response rate was 47% [37]. The benefit demonstrated in this study and subsequent ones led to FDA approval in 2022 of this treatment in any unresectable or metastatic locally advanced tumor that has received and progressed on standard first-line treatment [34]. EGFR is a transmembrane tyrosine kinase receptor involved in signaling pathways related to cell proliferation and apoptosis. It induces activation of the RAS/RAF/MAPK pathway and the Akt/mTOR (mammalian target of rapamycin) pathway. This is best known in non-small cell lung neoplasm in colorectal adenocarcinomas. Treatments used may include tyrosine-kinase inhibitors (e.g., osimertinib) and monoclonal antibodies (cetuximab or panitumumab) [38-40]. The first trial that studied the pathway involving EGFR in biliary carcinomas was called BINGO and was a phase 2, non-comparative study that enrolled patients with inoperable or metastatic stage, without previous treatment. They were allocated 1:1 to receive either gemcitabine 1000 mg/m2 and oxaliplatin 100 mg/m2 with or without cetuximab 500 mg/m2, repeated every two weeks until unacceptable toxicity or disease progression. The main end-point was the progression-free survival rate at four months. In the 76 enrolled patients, the median progression-free survival was 6.1 months (95% CI 5.1-7.6). Survival without progression was slightly longer in the group of patients without cetuximab (12.4 months versus 11.0 months). It was concluded that the addition of cetuximab to gemcitabine and oxaliplatin did not increase the effectiveness of chemotherapy in patients with advanced biliary carcinomas although the tolerance was relatively good, the study not changing the standard of therapy [41,42]. Another phase 2 study called Vecti-BIL verified the efficacy of panitumumab in combination with chemotherapy in patients with RAS wild-type, advanced bile duct carcinoma. A total of 89 patients, enrolled between June 2010 and September 2013, received gemcitabine and oxaliplatin (GEMOX regimen) with or without panitumumab for 12 cycles. No differences were observed between the two arms regarding progression-free survival (5.3 months in the arm that includes panitumumab and 4.4 months in the arm without) or overall survival. It was therefore obvious that panitumumab does not bring a benefit compared to chemotherapy alone in advanced bile duct carcinoma in the first line [43].

Immune checkpoint inhibitors for cholangiocarcinoma

The use of immune checkpoint inhibitors in metastatic cholangiocarcinoma is warranted in two clinical situations. The first refers to patients not selected by biomarker and is based on evidence from preclinical research identifying subgroups of cholangiocarcinoma with notable T-lymphocyte infiltration with antitumor activity [44]. It was considered that this aspect confers a positive prognosis and monotherapy with monoclonal antibodies against PD-1 (programmed cell death1) was further studied [45].

Keynote 158, a phase 2 trial, demonstrated reduced efficacy of pembrolizumab alone with an unselected population response rate of 5.8%, progression-free survival of two months, and median overall survival of 7.8 months. After these data, it was proposed to use immune checkpoint inhibitors for this clinical scenario in combination with cytotoxic chemotherapy [46].

The second clinical situation in which immune checkpoint inhibitors alone can be used in cholangiocarcinoma is the one in which biomarkers that have established this therapy in a tissue-agnostic regime are taken into account. In this regard, pembrolizumab is indicated in patients with high microsatellite instability or miss-match repair deficiency [47]. In the Keynote 158 phase 2 study, which looked at the use of pembrolizumab in patients with miss-match repair deficiency regardless of the primary tumor site, 22 patients had cholangiocarcinoma. In this cohort, pembrolizumab use was associated with a response rate of 40.8% and a complete response rate of 13.5%. Overall survival at three years was 30.3% [48]. This is the strongest evidence we have for using immune checkpoint inhibitors with notable efficacy.

## Conclusions

In conclusion, all the data summarized in the present review are evidence of the fact that NGS testing is of particular usefulness in cholangiocarcinoma. Bile duct cancers are rich in targetable genetic alterations, and their treatment is in constant change, although many of the current data comes from phase II studies. There is a great need for the current options to be analyzed in phase III studies. However, in countries where it is possible, the second-line treatment options are already based on the detection of the previously listed genetic alterations, with second-line chemotherapy remaining a reserve option. Hence, the need of the oncological community to stay informed about targeted treatment options in cholangiocarcinoma is supported by the present article.
